# Immunomodulatory Drugs in the Management of SARS-CoV-2

**DOI:** 10.3389/fimmu.2020.01844

**Published:** 2020-08-13

**Authors:** Daniel R. Burrage, Soraya Koushesh, Nidhi Sofat

**Affiliations:** ^1^Musculoskeletal Research Group, Institute for Infection and Immunity, St George's, University of London, London, United Kingdom; ^2^Department of Rheumatology, St George's University Hospitals NHS Foundation Trust, London, United Kingdom

**Keywords:** SARS-CoV-2, hyperinflammation, biologics, cytokines, immunomodulators

## Abstract

With the onset of the global pandemic in 2020 of Severe Acute Respiratory Syndrome Coronavirus-2 (SARS-CoV-2), there has been increasing research activity around certain disease-modifying drugs that are used for the management of inflammatory disorders such as rheumatoid arthritis, spondyloarthrosis, psoriatic arthritis, systemic lupus erythematosus, and inflammatory bowel disease for managing coronavirus symptoms. In the conditions mentioned, many people are on long-term treatment with agents including hydroxychloroquine, tumor necrosis factor alpha (TNFα) inhibitor drugs, other biologic agents such as monoclonal antibodies to IL-6 and Janus kinase inhibitors including baricitinib and tofacitinib, which are used to control inflammatory responses in their respective auto-immune condition. There is emerging data that immunomodulatory drugs could be protective at reducing certain features of SARS-CoV-2 and improving recovery. In addition, it is important to understand if subjects being treated with the immunomodulatory agents described have a less severe SARS-CoV-2 infection, as they are deemed some protection from their immunomodulatory treatment, or if they develop infections similar to non-immunocompromised patients. There is a huge unmet clinical need to advise patients responsibly about whether they should remain on their immunomodulatory treatment or not in light of Covid-19 infection. In this article we will discuss potential treatment options for SARS-CoV-2 using immunomodulatory drugs and at what stage of the condition they may be beneficial. Viable treatment options during the global coronavirus pandemic are a much-needed and an intensely active area of research.

## Introduction

The global pandemic of Severe Acute Respiratory Syndrome Coronavirus-2 (SARS-CoV-2), which originated in China in late 2019, has spread rapidly throughout the world to become a global pandemic. The emergence of this very infectious virus has placed huge burdens on populations worldwide, infecting millions and causing deaths in thousands of people across the globe. There is currently no cure for coronavirus. Although a diagnostic test is available for PCR testing of the virus by nasopharyngeal swab, there are cases in which clinical features are apparent, but a swab test may be negative, including cough, shortness of breath, temperature, often accompanied by laboratory changes such as lymphopenia, raised serum C-Reactive Protein (CRP), ferritin levels and pulmonary infiltrates on chest radiographs.

Coronavirus is primarily a respiratory illness affecting the lungs, which can lead to high temperatures, cough, headache, sore throat, shortness of breath, arthralgia, myalgia, chest pain, altered taste, and confusion. The condition can cause a rapid inflammatory response in the body, with the release of cytokines and acute deterioration. The coronavirus outbreak has led to new opportunities to study the immune response to coronavirus and to consider novel therapeutics for this condition.

Due to the lack of availability of a cure, there is a huge international effort to develop potential vaccines and pharmacotherapies to treat SARS-CoV-2. Among the candidate treatments, immunomodulatory agents have been proposed to target the inflammatory reaction that is induced in the lungs of affected patients and also the cytokine storm which affects people in severe cases. A number of agents more commonly used in inflammatory conditions, including corticosteroids, hydroxychloroquine, biologic inhibitors of IL-6 and IL-1, such as tocilizumab and anakinra respectively, TNFα inhibitors and janus kinase inhibitors have all been proposed as potential therapies for SARS-CoV-2 (1, 2), some of which are already in clinical trials, such as the RECOVERY trial (3). The RECOVERY trial, which is being co-ordinated in Oxford, has already recruited more than 11,500 participants from over 175 NHS hospitals in the UK and includes low dose corticosteroids, hydroxychloroquine, and tocilizumab, which are treatments commonly used to treat inflammatory arthritis (3). Other treatment arms in the trial include lopinavir-ritonavir, azithromycin, and convalescent plasma.

**Figure 1:**
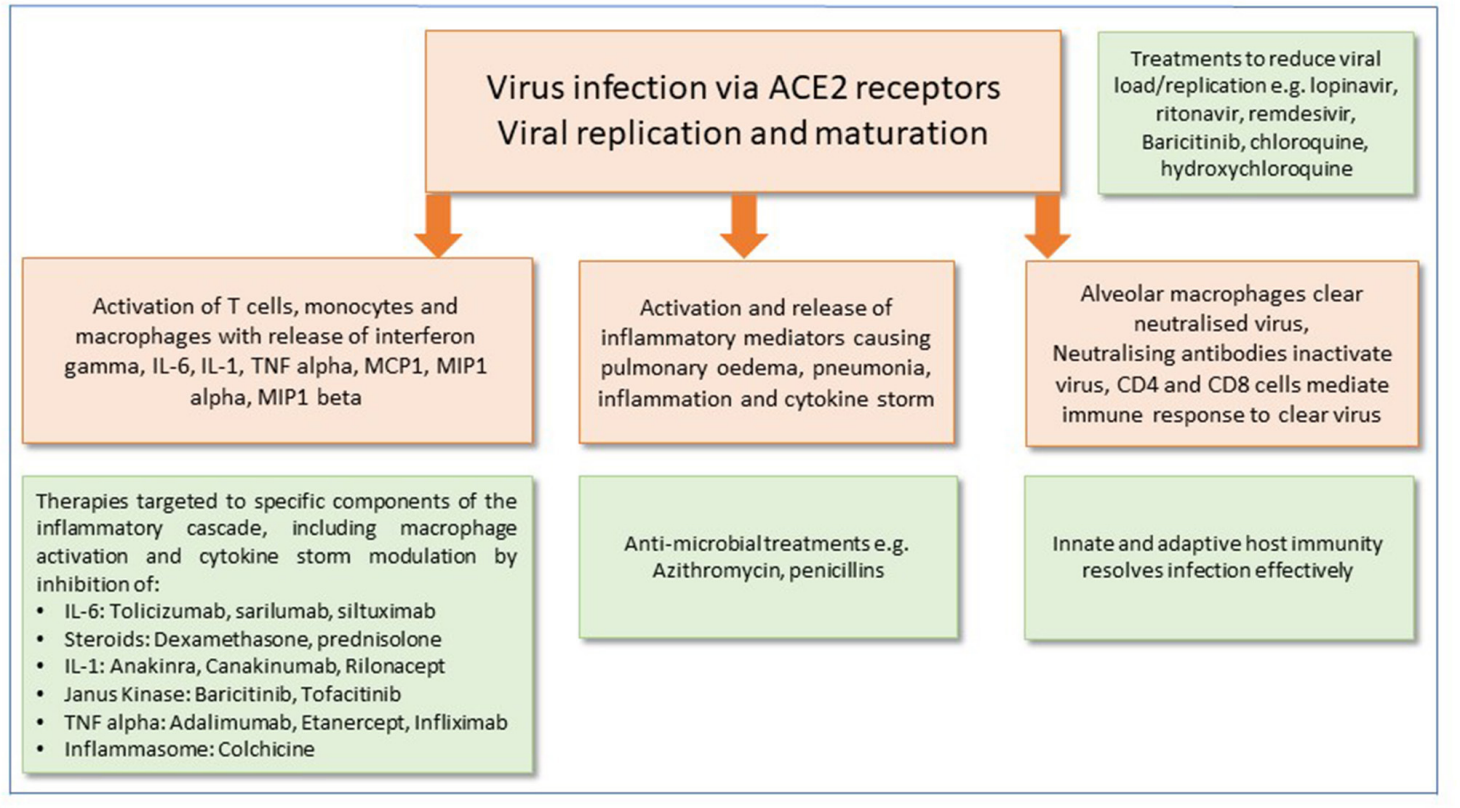
Potential therapeutic approaches for the management of SARS-CoV-2 infection. Mechanisms of cell injury and damage are shown in peach boxes. Green boxes show potential therapeutic targets and immune responses to modify and alleviate infection.

It is now apparent that SARS-CoV-2 infection has two clear clinical phases of infection: the former, which involves the viral infection and replication (4) and the inflammatory phase which often leads to rapid deterioration and worsening respiratory symptoms, requiring hospital admission in many cases to avoid deterioration (4, 5). Although corticosteroids are not routinely recommended and may exacerbate COVID-19-associated lung injury (4), in hyperinflammation, immunosuppression is likely to be beneficial. Mehta et al. reported features of a cytokine storm syndrome in a subgroup of COVID-19 patients (5). Further data indicate that an elevated ferritin (a hallmark feature of secondary hemophagocytic lymphohistiocytosis) and elevated IL-6 are predictors of fatality. It has been proposed that by screening for hyperinflammation to identify at risk groups, targeted immunomodulation could improve mortality (5). The current state of play of potential therapeutics that could be used to directly target the virus, or reduce its effects on the host response, are summarized in Figure 1.

In this review, we discuss the rationale for the potential use of immunomodulator therapies in the management of SARS-CoV-2. In particular, we will explore which patient subgroups with respect to infection severity and systemic response, the immunomodulators may be beneficial.

## Cytokine-Based Therapies

Targeted biologic therapies against specific cytokines have become the treatment of choice in active rheumatic inflammatory conditions. Over the last few decades, improved understanding of the immunology of inflammatory diseases, coupled with the advancement of technologies allowing mass production of biologic therapies, has transformed the management of conditions including rheumatoid arthritis, ankylosing spondylitis, and inflammatory bowel disease with cytokine-targeted biologic therapies.

Data from several groups has shown that cytokine levels are elevated in people hospitalized with SARS-CoV-2 infection, with a rapid release of cytokines such as IL-1, IL-6, and TNF alpha (3, 6–8). In the context of other concomitant risk factors such as male gender, increased age, immunocompromise, and obesity (9–13), rapid onset of the cytokine storm requires urgent treatment to prevent multi-organ failure and death.

It has been noted that severity of SARS-CoV-2 and increased deaths have been associated with several risk factors, including older age (9), male gender (10), black or minority ethnic origin (11), obesity (12), diabetes mellitus (13), and cardiovascular disease (9). Such observations have led to hypotheses that genetic risk factors for cytokine release syndrome (CRS) or cytokine storm (CS) may be at play. For example, conditions including Familial Mediterranean Fever (FMF) or TRAPS (Tumor Necrosis Factor Associated Periodic Fever Syndromes) are known to be more prevalent in specific ethnic groups, including Mediterranean, Arab, Jewish, Turkish, Armenian, North African descent with some mutations found in Asian populations.

Such observations, as highlighted above, have led to the concept that therapies targeted to IL-6, IL-1, and TNF alpha may have a role to play in the post-infection stage of SARS-CoV-2. In the post-infective stage, an accelerated inflammatory response sets in, which has important implications for the management of SARS-CoV-2 infection.

### IL-6 Cytokine Inhibitors

Biologics targeted to IL-6, such as tocilizumab, a humanized monoclonal antibody generated to the IL-6 receptor, are licensed for the management of active rheumatoid arthritis, juvenile idiopathic arthritis, and replasing or refractory giant cell arteritis (GCA) (6). They are also licensed for the treatment of cytokine release syndrome. IL-6 is a key cytokine in the mediation of fever and the acute phase reponse, including C-reactive protein and ferritin.

Tocilizumab has already been used in the context of severe Covid-19 infection. A recent retrospective study reported outcomes for 21 patients in China (7). Tocilizumab has been used in people with severe features of Covid-19, including in subjects with severe infection, having a respiratory rate ≥ 30 breaths/min, SpO_2_ ≤ 93% while breathing room air and a PaO_2_/FiO_2_ ≤ 300 mmHg. In this uncontrolled study, 21 patients with severe or critical Covid-19 pneumonia were treated with tocilizumab 400 mg intravenously (7). In many of the subjects treated, the fever returned to normal within a few days, 15 out of 20 lowered their oxygen requirement and one patient needed no further oxygen therapy. In 19 out of 20 subjects, there was an improvement in Computerized Tomography (CT) scans of the chest.

### Case History

We treated a case of severe Covid-19 in London in April 2020 with tocilizumab which was provided on a compassionate use basis. Compassionate drug use refers to use of this drug for an unapproved indication to treat seriously ill patients when no other treatments are available. A 54-year-old Kurdish woman attended the emergency department at St George's Hospital, London, with 7 days of gradually worsening headache, fever, a new productive cough and loss of taste.

The patient had a history of asthma (well-controlled with regular budesonide 200 micrograms and formoterol 6 mg combination inhaled twice a day and salbutamol 100 micrograms inhaled up to four times daily as required), subarachnoid hemorrhage (due to an anterior communicating artery aneurysm, managed with coiling), chronic headaches (managed with co-codamol 30/500 1–2 tablets up to four times daily as required, tramadol 50 mg orally twice daily and pregabalin 25 mg orally daily) and gastro-esophageal reflux disease (managed with lansoprazole 15 mg orally daily and sodium alginate with potassium bicarbonate 10 mL up to four times daily as required). She was a lifelong non-smoker, with no alcohol intake.

Her temperature was 37.8°C, heart rate 86 beats per minute, respiratory rate 18 breaths per minute, blood pressure 146/94 mmHg and peripheral oxygen saturation 99% breathing room air. On examination she had crepitations at the right lung base.

**Figure 2:**
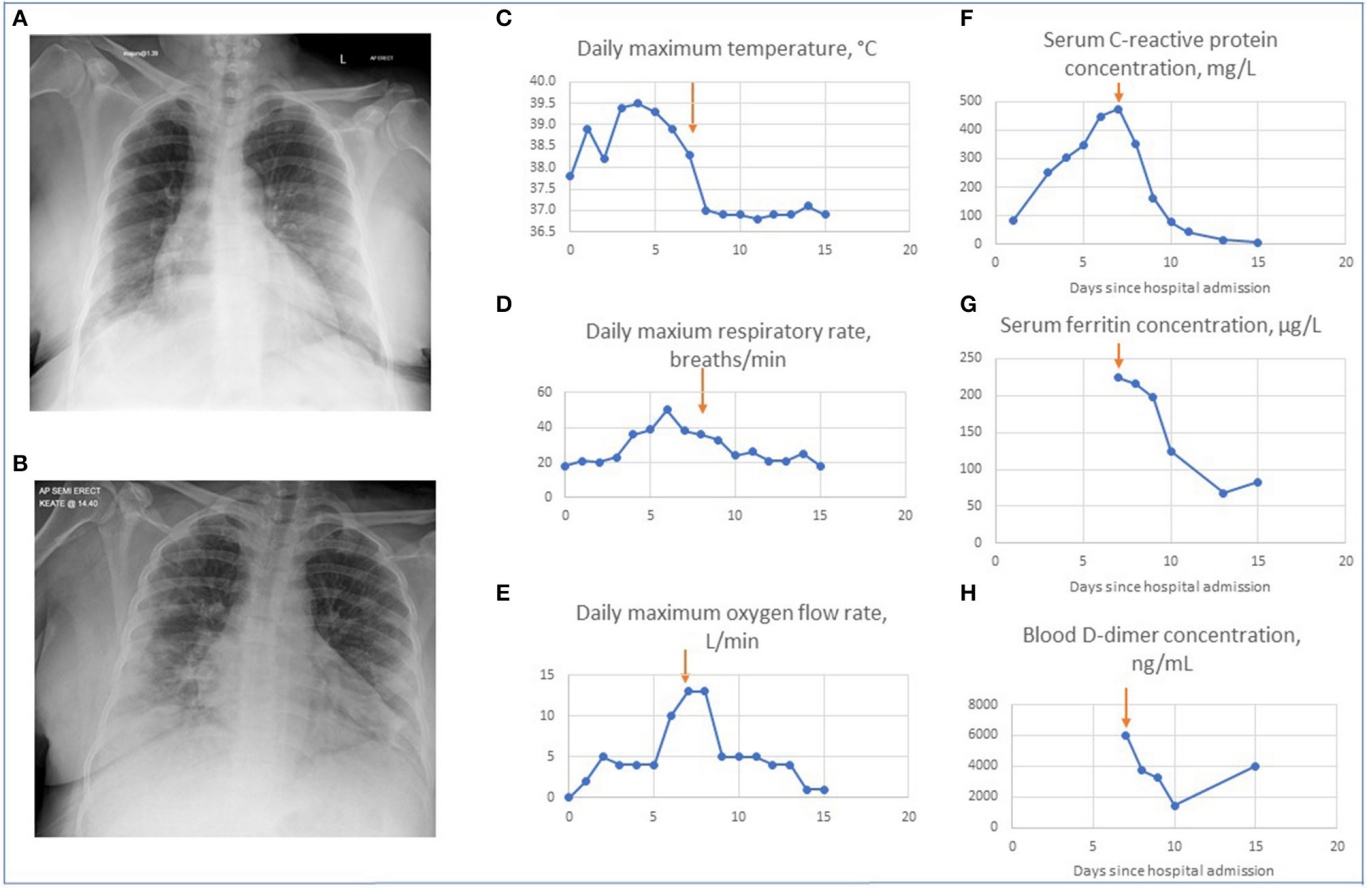
Changes in patient's clinical, radiographic and biochemical parameters on treatment with tocilizumab. **(A)** Chest radiograph on admission. This demonstrates minor bi-basal opacity more pronounced on the right. **(B)** Chest radiograph on day 3. This demonstrates patchy areas of consolidation within the lung peripheries bilaterally with retrocardiac left lower zone opacification with air bronchograms. Graphs showing changes in the maximal daily values of parameters for patient's hospital admission for temperature **(C)**, respiratory rate **(D)** oxygen requirement **(E)**, serum C reactive protein levels **(F)**, ferritin levels **(G)**, and D-dimers **(H)**. Arrows indicate Day 7 when tocilizumab was given.

Her blood neutrophil count was 7.8 × 10^9^/L, lymphocyte count of 0.9 × 10^9^/L and C-reactive protein 82 mg/L. Her chest radiograph demonstrated bi-basal opacities, more pronounced on the right-hand side (Figure 2). A respiratory swab was sent for SARS-CoV-2 RNA.

She was diagnosed with probable COVID-19 and initiated on doxycycline 200 mg once only, then to continue 100 mg daily for possible community acquired pneumonia. She was discharged with advice to self-isolate and a plan for review in the ambulatory medical care unit the next day.

On review the following day her peripheral oxygen saturations were 90% breathing room air with respiratory rate 17–21 breaths/min. Her nasopharyngeal swab detected SARS-CoV-2 RNA (Abbot Realtime SARS-CoV-2 assay). The Abbott RealTime SARS-CoV-2 assay is a real-time (rt) reverse transcriptase (RT) polymerase chain reaction (PCR) test used for the qualitative detection of nucleic acids from the SARS-CoV-2 in nasopharyngeal (NP) and oropharyngeal (OP) swabs from patients (14).

A decision was made to admit her for oxygen therapy, initially requiring 2 L/min oxygen to achieve oxygen saturations greater than 94%, and to continue doxycycline for possible supra-added bacterial pneumonia.

On day 3 of admission she continued to have fevers and felt increasingly breathless, with peak temperature 39.5°C, respiratory rate of 20–30 breaths per minute and oxygen requirement of 4 L/min. Her blood neutrophil count was 9.2 × 10^9^/L, blood lymphocyte counts 0.6 × 10^9^/L, and C-reactive protein 302 mg/L. A repeat chest radiograph showed patchy areas of consolidation within the lung peripheries bilaterally and retrocardiac left lower zone opacification with air bronchograms (Figure 2). Benzylpenicillin 1.2 g 4-hourly intravenously was commenced in addition to doxycycline to treat for possible supra-added severe pneumonia.

By day 7 of admission her work of breathing and oxygen requirements had continued to increase, with a respiratory rate of 32–38 breaths per minute and oxygen requirement of 13 L/min, with peak temperature 38.3°C. Her blood neutrophil count was 9.5 × 10^9^/L, blood lymphocyte counts 0.6 × 10^9^/L, C-reactive protein 474 mg/L, D-dimer >6,000 ng/mL, and ferritin 224 μg/L. A full and comprehensive infection screen was completed with no focus of bacterial infection: mycoplasma serology and urinary legionella and pneumococcal antigens were not detected and there was no bacterial growth on sputum, urine, or blood cultures. A decision was made to administer a single dose of intravenous tocilizumab, dosed at 8 mg/kg, accessed through a compassionate use off-license scheme, to treat for cytokine storm secondary to coronavirus infection, and she was reviewed for consideration of admission to critical care.

On day 8 of admission, within 12–24 h of tocilizumab administration, her oxygen requirements had reduced to 5 L/min and her respiratory rate had improved to 22–33 breaths per minute. Over the course of the following week she showed continuous improvement. By day 15 of admission she was discharged with respiratory rate of 18 breaths per minute and oxygen saturations 92% breathing room air, with no recorded fevers for 7 days. Two weeks after discharge her symptoms had continued to improve although she noted persistent loss of taste and occasional cough. Her imaging and improvement in biochemical parameters during her admission are shown in Figure 2. The clinical course of the disease and rapid response to treatment with tocilizumab suggested that our patient had cytokine storm secondary to SARS-CoV-2 infection, which was responsive to IL-6 inhibition.

Our case highlights that in severe Covid-19 infection, where subjects may be exhibiting features of cytokine storm with little reponse to full supportive care, there may be a case for treatment with a single dose of intravenous tocilizumab to reverse the effects of the cytokine storm and to prevent the positive feedback loop of release of pro-inflammatory cytokines which leads to rapid clinical deterioration and death in many cases.

There are several points to be noted from our case and in general from the pharmacology of tocilizumab treatment. Anti-IL-6 treatment often leads to a very rapid reduction in CRP levels as it is a strong suppressor of acute phase reactants produced by the liver. It is therefore important to monitor patients closely for other intercurrent infections e.g., bacterial, since they may fail to mount a full response as a result of inhibition of inflammatory pathways. Larger trials of IL-6 inhibitors for Covid-19 are now underway and will be important to establish the clinical scenarios in which it will be of optimal use (3, 8). Current data suggests that it may best be used in severely ill Covid-19 patients, to reduce the likelihood of subjects requring critical care or to prevent catastrophic cytokine storm features. In rheumatoid arthritis, IL-6 inhibitors are usually used as weekly or monthly injections. However, in the setting of acute Covid-19 related inflammation, a single dose may be adequate. Current trials are also testing repeated use in severe cases. In people with rheumatoid arthritis treated with IL-6 inhibitors, long-term monitoring for raised lipid levels and development of lower intestinal perforation are closely monitored, with patients who have underlying diverticular disease considered a relative contraindication for treatment in the rheumatoid arthritis setting. It remains to be seen whether in the acute infection setting of Covid-19 subjects are prone to the development of the side-effects previously observed for IL-6 inhibitors in other disease indications.

### IL-1 Cytokine Inhibitors

Interleukin-1 is a very active pro-inflammatory cytokine which is released during inflammatory processes including sepsis and chronic inflammation. It can lower pain thresholds but also cause sustained tissue damage (15). Monotherapy using the IL-1 receptor antagonist, anakinra, is already proven in several autoinflammatory syndromes including rheumatoid arthritis, hereditary systemic autoinflammatory diseases such as Familial Mediterranean Fever (FMF), Cryopyrin-associated periodic syndrome (CAPS), and TNF receptor-associated periodic syndrome (TRAPS). There are several commercially available inhibitors of IL-1 which are licensed, including the IL-1 receptor anatonist anakinra, the soluble decoy receptor rilonacept and the neutralizing monoclonal antibody to IL-1 beta, canakinumab.

IL-1 modulators are often extremely effective in conditions where there are sustained fevers and a marked systemic inflammatory response. For example, we recently treated a case of unexplained fevers, weight loss and night sweats, in a patient with no known infection, who had genetic sequencing that showed a mutation in intron 4 of the gene for TNF receptor superfamily 1A (TNFRSF1A), c.473-72 G > A, which demonstrated the diagnosis of tumor necrosis factor-associated periodic fever syndrome (TRAPS) (16). Our patient underwent treatment with the IL-1 receptor antagonist anakinra at 100 mg daily subcutaneously and within 2 days he had symptomatic improvement, suppression of CRP and serum amyloid A levels began to normalize (16).

There are centers across the world that are currently using anakinra for CRS and CS-related features of Covid-19. The importance of release of IL-1 and IL-6 pro-inflammatory cytokine released by lung tissue in response to toll-like receptor activation during SARS-CoV-2 infection is recognized and are valid treatment targets (17). It remains to be seen if there are specific clinical differences in outcome between IL-1 or IL-6 inhibition in the setting of severe SARS-CoV-2 infection. It may be that IL-6 inihibitors may be preferred as a single injection that has sustained effect over a longer period of time, in comparison to IL-1 inhibitors, which since they are usually given as a daily injection and therefore may require repeated dosing.

### TNF Alpha Cytokine Inhibitors

The advent of biologic therapies targeted at the inhibition of TNFα in the 1990s led to a step change in the management of many inflammatory conditions for which the drugs are licensed, including rheumatoid arthritis, ankylosing spondylitis, psoriatic arthritis, juvenile arthritis, and inflammatory bowel disease. Currently a wide variety of formulations of TNF inhibitors are used, including fully humanized biologics targeted to TNFα that include adalimumab, etanercept, and infliximab. The demonstration that TNFα is a key cytokine that is produced in a wide range of conditions causing inflammation, both in the acute and chronic phase, has been borne out by its success as a treatment in a broad range of conditions. In conditions such as rheumatoid arthritis, blockade of TNFα leads to a subsequent decrease in IL-1 and IL-6, adhesion molecules and angiogenic factors such as vascular endothelial growth factor (VEGF). The rationale for the use of TNF inhibitors in hospitalized patients with SARS-CoV-2 has been proposed (18). In people with inflammatory arthritis and inflammatory bowel disease, screening for tuberculosis (TB) and malignancy are performed and subjects with a history of latent or active TB are commenced on TB eradication treatment before starting TNF inhibitors. In addition, people with a cancer history within the previous 5 years are not usually given TNF inhibitors. Such considerations may be overriden in the acute setting of infection with Covid-19, but may have long-term consequences and should be considered in study designs.

## Corticosteroids

Corticosteroids have the ability to suppress inflammation by acting on reducing the activation of several inflammatory mediators produced by the body during infection and inflammation (19). Corticosteroids bind to a corticosteroid receptor (CR) and the complex translocates to the nucleus where it binds to the glucocorticoid response element (GRE). This complex increases the transcription of a number of anti-inflammatory genes, including those encoding inhibitory (I)-κB, which inhibits the activation of nuclear factor (NF)-κB, genes encoding cytokines IL-4, IL-10, IL-13, and TGFβ (19, 20). The corticosteroid-CR complex inhibits binding of transcription factors (AP)-1 and (NF)-κB to their response elements, thereby reducing the production of pro-inflammatory cytokines IL-1β and TNFα in activated macrophages. Corticosteroids also increase the synthesis of lipocortin-1, which inhibits the precursor of eicosanoids, platelet activating factor and phospholipase A_2_. The multiple mechanisms of action of glucocorticoids make them effective at suppressing inflammatory responses at several sites, including the lung tissue, joint, and systemic inflammation.

Infection with SARS-CoV-2 infection induces destruction within lung cells, which triggers a local immune response by activation of macrophages and monocytes, cytokine release and induce T and B cell responses. The innate and adapative immune response is usually sufficient to clear the virus-induced damage in most cases. However, in some people an altered immune reponse occurs, with development of severe lung and systemic pathology. Due to their effects on multiple aspects of inflammation, corticosteroids can be used in the early stages of cytokine storm and macrophage activation syndrome (MAS), when there is an overwhelming inflammatory response in the body, often in response to an infectious trigger. Several studies have shown there is a positive effect by corticosteroids in reducing immunopathological damage (21). However, other studies have shown that viral RNA concentrations of SARS-CoV-2 can increase with corticosteroid treatment compared with placebo (22). It may be more prudent to use corticosteroids in a peri-intensive care setting, when subjects may be entering a cytokine storm, rather than in treating ambulatory patients or those only requiring routine care for their infection. Indeed initial analysis from the RECOVERY trial of 2,104 patients randomized to receive dexamethasone 6 mg once per day fro 10 days (orally or intravenously) has demonstrated a reduction in 28-day mortality in ventilated patients and patients requiring oxygen compared to those receiving usual care (23) There was no benefit for patients who did not require respiratory support. Peer review publication of this data is awaited.

## Chloroquine and Hydroxychloroquine

Chloroquine and hydroxychloroquine are used widely across the world as antimalarials. They also have a role in the treatment of systemic lupus erythematosus (SLE), rheumatoid arthritis (RA) and other inflammatory rheumatic diseases. Chloroquine and hydroxychloroquine are weak bases (24). They have a broad volume of distribution and a half-life of approximately 50 days (25). They have multiple mechanisms of action, including altering cell pH, affecting lysosomal activity, autophagy, signaling pathways, and inhibition of cytokine production and co-stimulatory molecules (26). Recently, chloroquine was identified as having potent activity against SARS-CoV-2 (27). Although clinical trials of chloroquine and hydroxychloroquine are currently underway in the treatment of SARS-CoV-2, there are questions that currently remain unanswered. These include the optimal timing of using the drug; some reports suggest early use to inhibit viral replication may be optimal, whereas several clinical trials are using chloroquines at high dose for patients with symptoms severe enough to require hospital admission (3), ranging from 500 to 1,000 mg per day. The potential longer-term toxicity effects of the chloroquines in the context of SARS-CoV-2, e.g., myocarditis, arrythmias, retinal toxicity, are not known in the context of randomized controlled trials (28). However, emerging data from the RECOVERY trial from a total of 1,542 patients randomized to hydroxychloroquine compared to 3,132 patients randomized to usual care has not shown hydroxychloroquine to be effective in reducing mortality or hospital stay duration (29).

## Janus Kinase Inhibitors

The Janus kinase inhibitors (JAKis) are also known as targeted synthetic disease-modifying anti-rheumatic drugs (tsDMARDs) (30). JAKis block cytokine signaling by inhibiting the phosphorylation of activated cytokine receptors. When activated, the phosphorylated cytokine receptors recruit STAT transcription factors which modulate gene transcription.

They are currently the only licensed tsDMARDs for the management of active rheumatoid arthritis. Drugs included in the JAKi group include baricitinib, tofacitinib, perficitinib, filgotinib, upadacatinib, and fostaminib. In the management of RA, a safety signal reported has been the increased risk of herpes zoster infection, especially in Japanese and Korean patients with RA (31). It has been argued that JAKis may not be useful in the early stages of infection with SARS-CoV-2, since the activity of interferons, which are often the major mediators of viral clearance in the body, are mediated via the JAK-STAT signaling pathway. JAKis have been proposed as a treatment in severe coronavirus infection with features akin to cytokine storm (32). Recently, an open-label study testing the effect of baricitinib was published of 12 patients in Italy treated for SARS-CoV-2 infection (33). There were 10 males and 2 females in the study group, with a mean age of 63.5. Fever, oxygen saturations, oxygen requirements and C-reactive protein significantly improved in the baricitinib group compared with controls. A transfer to the Intensive Care Unit was requested in 33% (4/12) of controls and in none of the baricitinib-treated patients (*p* = 0.093). Discharge at week 2 occurred in 58% (7/12) of the baricitinib-treated patients vs. 8% (1/12) of controls (*p* = 0.027). However, this small trial of 12 subjects was open-label and not randomized. Larger randomized controlled trials are now underway to assess the value of baricitinib in the management of SARS-Cov-2 infection. Several clinical trials are underway of baricitinib therapy in comparison to anti-viral therapies (NCT04320277 and NCT04321993), but have not reported so far. In a recent study reported from the USA in 86 subjects who developed SARS-CoV-2 and also had an immune-mediated inflammatory condition, 62% of subjects were on a biologic drug or JAKi, but of those only 7% of those were hospitalized (34). The US case series data in people who developed SARS-CoV-2 suggests that being on an immunomodulator did not appear to increase the risk of developing SARS-CoV-2 features that led to serious infection or death in this case series.

## Inflammasome

Colchicine is a microtubule inhibitor drug widely used in the management of gout and conditions that involve localized inflammation including serositis e.g., Behcet's disease, Systemic Lupus Erythematosus (SLE), and pericarditis (35, 36).

Myocardial injury is recognized in SARS-CoV-2 infection, with an imbalance of oxygen supply and demand due to Adult Respiratory Distress Syndrome (ARDS) and acute lung injury. Histologically proven myocarditis has been found in SARS-CoV-2 infection, and the additional injury caused to cadiac tissue by activation of a cytokine storm, with vascular inflammation, endothelial dysfunction, and arrhythmias have been observed (37). It has been suggested that the NLRP3 inflammasome activation, which is initiated by viroporin E, is a component of SARS-CoV-2 (38), thereby inducing an inflammatory response. Since colchicine has been shown to inhibit the NLRP3 inflammasome (39), it is a potential valid target for the use of colchicine in Covid-19 infection. There are already 4 clinical trials announced that will be investigating the use of colchicine in SARS-CoV-2 with endpoints including need for hospitalization or death. Some trials are designed as colchicine monotherapy in addition to standard clinical care (ClinicalTrials.gov Identifier: NCT04322682, ClinicalTrials.gov Identifier: NCT04326790, ClinicalTrials.gov Identifier: NCT04322565), whereas other trials are designed with concomitant administration of anti-viral therapy including lopinavir/rotinavir (ClinicalTrials.gov Identifier: NCT04328480).

## Conclusions

Our review has discussed the wide range of clinical features with which SARS-CoV-2 infection can present. Recognizing which clinical features are most likely to be targeted by specific therapies will be crucial to establish optimal therapeutics for treating infection. For example, anti-viral agents may be needed to target prevention of viral entry and replication, whereas immunomodulatory drugs are most likely to play a role in cytokine storm and macrophage activation in patients who are at high risk of requiring intensive care in order to prevent uncontrolled inflammation and death. There is a huge need to conduct well-designed, randomized controlled trials in the context of SARS-CoV-2 infection, so that true signal outcomes for efficacy are determined that lead to evidence-based therapies for the global pandemic.

## Author Contributions

NS conceived and wrote the manuscript. SK collated references and assisted in writing the manuscript. DB wrote the case history in the manuscript and managed the patient with NS. All authors contributed to the article and approved the submitted version.

## Conflict of Interest

The authors declare that the research was conducted in the absence of any commercial or financial relationships that could be construed as a potential conflict of interest.
